# Rectal Medication by Means of Gases

**Published:** 1887-04

**Authors:** 


### EDITORIAL.

RECTAL MEDICATION BY MEANS OF GASES,

Though not absolutely new, has been prominently brought to the notice of the profession by Professor Bergeon. His experiments have been repeated by quite a large number of persons both in Europe and in this country. It is too soon to pronounce positively as to the value of the method, but so far there seems to be but little danger of doing harm, though a few cases have occurred in which there were developed disagreeable symptoms. The method consists in injecting into the rectum sulphuretted hydrogen mixed with carbonic acid. The proportion of the sulphuretted hydrogen should be small. The gas is taken up by the veins of the lower alimentary canal and eliminated through the lungs. As it is an antiseptic it acts upon the morbid structures through which it escapes. Such is the theory. So far as known, it does not seem to be a bactericide.

Cases now under treatment in this city are certainly improving, for some reason. The temperature is diminishing, night-sweats and expectoration are less, and their general condition is more comfortable.

The apparatus devised by Morel, in Paris, has been prepared in Chicago, and can be used easily by anyone who may desire to test this method. The carbonic acid is liberated by the action of sulphuric acid upon a carbonate; as, for instance ordinary carbonate of soda. The sulphuretted hydrogen is, by Bergeon, combined with the acid by passing this latter gas through the natural sulphuretted waters. The mixture, contained in any convenient form of gas-holder, is ready for use. It should be introduced into the rectum unmixed with atmospheric air, as this latter may produce griping.

